# Status of cardiovascular health in the Republic of Serbia: Results from the National Health Survey

**DOI:** 10.1371/journal.pone.0214505

**Published:** 2019-03-27

**Authors:** Janko Janković, Maša Davidović, Vesna Bjegović-Mikanović, Slavenka Janković

**Affiliations:** 1 Institute of Social Medicine, Faculty of Medicine, University of Belgrade, Belgrade, Serbia; 2 Centre—School of Public Health and Management, Faculty of Medicine, University of Belgrade, Belgrade, Serbia; 3 Institute of Epidemiology, Faculty of Medicine, University of Belgrade, Belgrade, Serbia; University of Bologna, ITALY

## Abstract

**Background:**

Cardiovascular diseases (CVD) remain the most significant cause of death in low- and middle-income countries where the burden of CVD continues to rise due to the increasing incidence of CVD. The aim of this study was to assess the prevalence of ideal cardiovascular health (CVH) across sex and age groups and to analyze associations between demographic and socio-economic variables and ideal CVH metrics in the adult population of the Republic of Serbia.

**Methods:**

Information on demographic (age, sex, marital status, and type of settlement) and socio-economic characteristics (education, and wealth index), and the 7 ideal CVH metrics (smoking, physical activity, healthy diet, BMI, blood pressure, cholesterol, and glucose) was obtained for 13100 respondents aged 20 years and above, from the 2013 National Health Survey in the Republic of Serbia. According to the American Heart Association, the ideal CVH was defined as the simultaneous presence of 7 ideal CVH metrics.

**Results:**

Out of all ideal CVH metrics, the most prevalent components were ideal glucose (92.2%), ideal cholesterol (86.4%) and ideal smoking (63%), while the least prevalent ideal CVH component was ideal healthy diet (2.4%). Only 0.1% had all 7 CVH components at the ideal level. According to the multivariable logistic regression analysis the higher number of ideal CVH metrics was observed in women (OR = 4.46), younger people (OR = 7.12), people living without partner (OR = 1.70), more educated (OR = 2.51 for middle educated and OR = 3.57 for high educated), as well as among the rich (OR = 1.43).

**Conclusion:**

Our findings of existing age-specific, sex and socio-economic differences in the prevalence and number of ideal CVH metrics should serve for the development of appropriate CVD prevention policies tailored to fit specific needs of both sexes, all age groups and people with different socioeconomic status.

## Introduction

Cardiovascular diseases (CVD) are the world’s biggest killer [1], accounting for 17.7 million deaths every year, that is 31% of all global deaths [2]. In recent decades the declining trends in CVD mortality rates have been recorded in high-income countries of the world, while CVD remain the most significant cause of death in low- and middle-income countries where the burden of CVD continues to rise due to the increasing incidence of CVD [2,3].

The Republic of Serbia is an upper-middle-income country situated in the Balkan Peninsula in Southeastern Europe and has been suffering substantial economic and health care system crisis in the last 30 years. In 2014, CVD were responsible for 54% of all causes of death in Serbia [4], and the standardized CVD mortality rates were 991 per 100,000 men and 836 per 100,000 women in 2013 [5].

Ideal cardiovascular health (CVH), as defined in the 2010 American Heart Association's national goal [6], describes a construct of 7 CVH metrics, namely smoking, physical activity, healthy diet, body mass index (BMI), blood pressure, blood cholesterol and glucose at ideal levels. There is increasing evidence that ideal CVH is inversely associated with cardiovascular events and CVD mortality [7–9]. Recently done meta-analysis showed a strong inverse linear dose-response relationship between ideal CVH metrics and CVD mortality, suggesting that even modest improvements in CVH is associated with substantial mortality benefit [10].

Considering that CVD incidence is increasing in low- and middle-income countries, and that the AHA CVH construct is a simple and cost-effective tool to assess and monitor CVH at the population level, assessing CVH in these countries should be a priority [11,12].

The aim of this study was to assess the prevalence of ideal CVH across sex and age groups and to analyze associations between demographic and socio-economic variables and ideal CVH metrics in the adult population of the Republic of Serbia.

## Method

### Study design and sample

This cross-sectional study used data from the 2013 National Health Survey (NHS) for the population of Serbia designed to ensure a nationally representative sample of the Serbian population. People living in collective households and/or institutions, and residents of Kosovo and Metohia (under the United Nations Mission) were excluded from the survey.

The NHS was performed in line with the EUROSTAT recommendations [13]. A stratified, two-staged representative random sample of the Serbian population was used. In total, 670 Census enumeration areas were defined as primary sampling units, by probability proportional to size sampling procedures. In the 16474 registered households, as the units of the second stage of sampling, 14623 members aged 15 years and above were interviewed. For the purpose of this study, our analysis was limited to the respondents aged 20 years and above, free of CVD (n = 13100) out of which 11746 respondents provided all necessary data for the calculation of 7 ideal CVH metrics. Participants excluded from the analysis did not differ statistically significant from those included. More details about the 2013 NHS are available elsewhere [14].

Information on demographic and socio-economic characteristics, and self-reported CVH metrics was obtained by face-to-face interview using a standardized questionnaire, while information on BMI and blood pressure was gathered by anthropometric and blood pressure measurements, respectively. All interviews and all measurements were carried out at the participant's home by trained staff.

Written informed consent was obtained for all participants. The study was approved by the Ethics Review Board of the Institute of Public Health of Serbia (Decision number 5996/1, of October 1, 2013).

### Study variables

The following demographic variables were selected from the database: age categorized into three groups (young: 20–39, middle age: 40–64 and older age: ≥65), sex, marital status (married/living with a partner or living without a partner), and type of settlement (urban/rural). Two socio-economic variables were analyzed: education defined as low (no education, incomplete primary school and primary school), middle (three or four years of secondary school), and high (college or university education) and Demographic and Health Survey Wealth Index (hereafter: wealth index) as a proxy for household wealth. According to the wealth index, respondents were classified into three wealth index groups: poor, middle, and rich. Variables related to the possession of examinees’ assets, i.e. household facilities were used in the wealth index calculation: number of bedrooms per household member, main material used for floor, roof, and walls of house, main source of drinking water and sanitation, source of energy used for heating, and possession of colour TV, mobile phone, refrigerator, computer, washing machine, dishwasher, air conditioning, central heating, car, and access to the internet. For the assignment of weights or factor scores to each variable, factor analysis and principal components analysis (PCA) were used with Varimax orthogonal rotation. Details about the wealth index calculation can be found elsewhere [15,16].

### Assessment of cardiovascular health (CVH)

The 7 CVH metrics (smoking, physical activity, healthy diet, BMI, blood pressure, cholesterol, and glucose), relevant NHS questions for self-reported metrics, and definitions for ideal CVH metrics are shown in Table 1.

**Table 1:** Criteria for ideal cardiovascular health metrics.

CVH metric	Question/measurement	Defininition for ideal CVH*
Smoking	Have you ever smoked? Do you smoke now?	Answered “no” on both questions
Physical activity	How much time do you spend walking in order to get to and from places on a typical day? How much time do you spend bicycling in order to get to and from places on a typical day?	Answered: ≥150 minutes a week of moderate activity
Healthy diet	How many portions of fruit do you eat each day? How many portions of vegetables and salad, excluding juice and potatoes, do you eat each day? How often do you eat fish? Which type of bread is most commonly used in your diet? Do you add salt to food you eat?	3−4 ideal components (fruits and vegetables ≥4–5 portions per day; fish ≥2 times per week; integral bread ≥3 times per day; added salt to food: “no”)
Body mass index (BMI)	Measured values of body weight and height	BMI (kg/m^2^) <25.0
Blood pressure	Measured values of SBP/DBP Do you treat high blood pressure?	SBP/DBP <120/80 mm Hg, untreated (answered “no” on question about treatment)
Cholesterol	Have you had high blood cholesterol in the past 12 months?	Answered “no”
Glucose	Have you had diabetes in the past 12 months?	Answered “no”

*Acording to American Heart Association

SBP: systolic blood pressure; DBP: diastolic blood pressure.

Smoking status, physical activity, healthy diet, cholesterol and glucose were determined by self-reported information (Table 1). Ideal smoking was defined as never smoked or stopped smoking for more than one year. The healthy diet was based on 4 out of 5 original AHA components (intake of fruits and vegetables, fish, whole grains, and sodium). For each component at an ideal level, the person got 1 point. Ideal healthy diet was determined if the person had a total score of 3–4 points. Ideal physical activity based on responses to relevant questions was defined as: ≥150 minutes a week of moderate activity (Table 1). Because blood cholesterol and blood glucose levels could not be directly measured due to the lack of financial resources, high cholesterol and diabetes were categorized as “no” (ideal) or “yes” based on the self-reported responses (Table 1).

Weight and height were measured with participants wearing light clothing and no shoes, using a validated electronic medical scale and portable wall-mounted stadiometer. BMI was calculated as weight in kilograms divided by squared heights in meters [17]. Blood pressure was measured using standardized procedures [18] with validated automatic devices and cuffs of 3 sizes according to the arm. Blood pressure was measured 3 times at 1–2 minutes interval and it was calculated as the mean of all 3 readings.

According to the AHA [6], the ideal CVH was defined as the simultaneous presence of 7 ideal CVH metrics: 4 ideal health behaviors (smoking, physical activity, healthy diet, and BMI) and 4 ideal health factors (blood pressure, cholesterol, glucose, and smoking). To emphasize the importance of smoking to health promotion the AHA Committee includes smoking in the both lists of factors [6]. In the present study poor CVH was defined as the presence of 0–1 ideal CVH metric.

### Statistical analysis

Continuous variables were described with means and standard deviations while categorical ones with frequencies and percentages. Prevalence rates with appropriate 95% confidence intervals (CIs) for ideal CVH, ideal health behaviors, and ideal health factors were estimated for the total population of Serbia, separately for men and women and also for young, middle-aged and older adults. In order to assess the differences between groups the chi-square test, t-test and one-way ANOVA were used where appropriate. Pooled proportions were estimated for the prevalence of ideal CVH metrics, the frequency of persons achieving 0, 1, 2, 3, 4, 5, 6 and 7 ideal CVH metrics, and in the following categories: 0 to 1, 2 to 5, and 6 to 7 ideal CVH metrics.

Associations between ideal CVH metrics and demographic (sex, age, marital status, type of settlement) and socio-economic variables (education and wealth index) were analysed with multivariable logistic regression analyses. The dependent variables formed three different models (first: 6–7 vs. 0–5 ideal CVH metrics; second: 3–4 vs. 0–2 ideal health behaviors; and third: 4 vs. 0–3 ideal health factors). The reason for using different cut-points for ideal behaviors and ideal health factors is a small number (0.4%) of people with all 4 ideal health behaviors. Statistical significance was set at 2-sided P <0.05. All statistical analyses were performed using SPSS version 20.0 software (SPSS Inc., Chicago, IL, USA) and STATA version 11.1 (StataCorp LP College Station, TX, USA).

## Results

The present study included in total 13100 adults aged ≥20 years, out of which more women (54.3%) than men (45.7%).

Characteristics of survey participants across sex and age groups were presented in Table 2. The average age of participants was 51.0 ± 17.4 years. Most of them were urban dwellers, married or living with a partner, had a middle education and belonged to poor wealth index group. Positive history of high blood cholesterol, diabetes and smoking were reported by 13.7%, 8.1%, and 50.3%, respectively.

**Table 2:** Characteristics of survey participants across sex and age groups, Republic of Serbia, 2013.

Characteristics	All (n = 13100)	Women (n = 7115)	Men (n = 5985)	P*	Age 20–39 (n = 3924)	Age 40–64 (n = 6039)	Age ≥65 (n = 3137)	P*
Age, mean (SD)	51.0 (17.4)	51.7 (17.6)	50.1 (17.1)	<0.001	30.0 (5.8)	52.8 (7.2)	73.9 (6.3)	-
Type of settlement, n (%)				0.019				<0.001
Urban	7408 (56.5)	4090 (57.5)	3318 (55.4)		2314 (59.0)	3404 (56.4)	1690 (53.9)	
Rural	5692 (43.5)	3025 (42.5)	2667 (44.6)		1610 (41.0)	2635 (43.6)	1447 (46.1)	
Education, n (%)				<0.001				<0.001
Low	3704 (28.3)	2460 (34.6)	1244 (20.8)		457 (11.6)	1509 (25.0)	1738 (55.4)	
Middle	7169 (54.7)	3496 (49.1)	3673 (61.4)		2674 (68.1)	3530 (58.5)	965 (30.8)	
High	2227 (17.0)	1159 (16.3)	1068 (17.8)		793 (20.2)	1000 (16.6)	434 (13.8)	
Marital status, n (%)				<0.001				<0.001
Married/living with partner	8573 (65.4)	4449 (62.5)	4124 (68.9)		2075 (52.9)	4761 (78.8)	1737 (55.4)	
Living without partner^†^	4527 (34.6)	2666 (37.5)	1861 (31.1)		1849 (47.1)	1278 (21.2)	1400 (44.6)	
Wealth index group, n (%)				0.127				<0.001
Poor	5700 (43.5)	3039 (42.7)	2661 (44.5)		1367 (34.8)	2586 (42.8)	1747 (55.7)	
Middle	2609 (19.9)	1443 (20.3)	1166 (19.5)		800 (20.4)	1255 (20.8)	554 (17.7)	
Rich	4791 (36.6)	2633 (37.0)	2158 (36.1)		1757 (44.8)	2198 (36.4)	836 (26.6)	
BMI, kg/m^2^, mean (SD)	26.7 (5.0)	26.5 (5.5)	26.9 (4.3)	<0.001	24.6 (4.6)	27.6 (5.0)	27.6 (4.9)	<0.001
SBP, mmHg, mean (SD)	136.3 (21.2)	134.8 (22.6)	138.2 (19.2)	<0.001	123.9 (13.2)	137.2 (19.6)	150.1 (23.1)	<0.001
DBP, mmHg, mean (SD)	82.1 (11.2)	80.5 (11.2)	84.1 (10.8)	<0.001	77.7 (9.4)	84.4 (10.9)	83.3 (12.0)	<0.001
History of high cholesterol, n (%) Yes No	1794 (13.7) 11306 (86.3)	1156 (16.2) 5959 (83.8)	638 (10.7) 5347 (89.3)	<0.001	110 (2.8) 3814 (97.2)	1059 (17.5) 4980 (82.5)	625 (19.9) 2512 (80.1)	<0.001
History of diabetes, n (%) Yes No	1067 (8.1) 12033 (91.9)	625 (8.8) 6490 (91.2)	442 (7.4) 5543 (92.6)	0.004	37 (0.9) 3887 (91.9)	491 (8.1) 5548 (91.9)	539 (12.2) 2598 (82.8)	<0.001
History of smoking, n (%) Yes No	6590 (50.3) 5534 (42.2)	3081 (43.3) 3477 (48.9)	3509 (58.6) 2057 (34.4)	<0.001	2105 (53.6) 1641 (41.8)	3492 (57.8) 2206 (36.5)	993 (31.7) 1687 (53.8)	<0.001

*According to chi-square test/t-test/one-way ANOVA where appropriate.

^†^Unmarried, divorced or widowed.

BMI, Body mass index; SBP, Systolic blood pressure; DBP, Diastolic blood pressure.

Compared with men, women were slightly older, less educated, more frequently lived without partner, had lower BMI, lower levels of blood pressure and more frequently reported a history of blood cholesterol and diabetes, while men smoked more often (Table 2).

Young people were more likely to be from urban areas than older ones, with higher education, without a partner, belonged to the rich wealth index group, and had lower BMI values, as well as, levels of blood pressure, while less frequently indicated presence of high cholesterol and diabetes. The smokers were mostly from the middle age group, while the lowest smoking rates were among the oldest individuals (Table 2).

Out of all ideal CVH metrics in the Serbian population, the most prevalent components were ideal glucose (92.2%), ideal cholesterol (86.4%) and ideal smoking (63%), while the least prevalent ideal CVH component was ideal healthy diet with only 2.4% (Table 3).

**Table 3:** Prevalence of ideal cardiovascular health metrics by sex and age groups, Serbia, 2013.

CVH metrics	All (n = 11746) % (95% CI)	Women (n = 6341) % (95% CI)	Men (n = 5405) % (95% CI)	P*	Age 20–39 (n = 3655) % (95% CI)	Age 40–64 (n = 5535) % (95% CI)	Age ≥65 (n = 2556) % (95% CI)	P*
Ideal smoking	63.8 (62.9–64.7)	66.7 (65.5–67.9)	60.4 (59.1–61.7)	<0.001	57.0 (55.5–58.5)	58.8 (57.6–60.1)	84.2 (82.4–86.0)	<0.001
Ideal healthy diet	2.4 (2.1–2.7)	2.9 (2.5–3.2)	1.8 (1.4–2.2)	<0.001	1.6 (1.1–2.1)	2.5 (2.1–2.9)	3.2 (2.7–3.8)	<0.001
Ideal physical activity	52.7 (51.8–53.7)	48.2 (47.0–49.4)	58.1 (56.8–59.4)	<0.001	57.2 (55.6–58.8)	55.8 (54.5–57.1)	39.8 (37.9–41.7)	<0.001
Ideal BMI	40.4 (39.5–41.3)	45.2 (44.0–46.4)	34.7 (33.4–36.0)	<0.001	59.3 (57.8–60.9)	32.7 (31.5–33.9)	29.9 (28.1–31.8)	<0.001
Ideal blood pressure	17.5 (16.9–18.2)	24.6 (23.7–25.5)	9.3 (8.3–10.3)	<0.001	34.3 (33.1–35.4)	12.6 (11.6–13.5)	4.4 (3.0–5.8)	<0.001
Ideal cholesterol	86.4 (85.7–87.0)	83.7 (82.9–84.6)	89.4 (88.5–90.3)	<0.001	97.2 (96.1–98.3)	82.6 (81.7–83.4)	79.1 (77.8–80.4)	<0.001
Ideal glucose	92.2 (91.7–92.6)	91.5 (90.8–92.2)	92.9 (92.2–93.6)	0.004	99.1 (98.2–99.9)	92.1 (91.5–92.8)	82.3 (81.3–83.3)	<0.001

CVH, Cardiovascular health

BMI, Body mass index

*According to Chi-square test.

While women were more likely to have ideal smoking, ideal BMI, ideal blood pressure and ideal healthy diet, other ideal CVH metrics were more common in men. These differences were highly statistically significant (Table 3).

Regarding age group, the prevalence of CVH metrics at the ideal level, except a healthy diet, was the highest among the youngest individuals. On the other side, the ideal healthy diet was the most prevalent in the oldest age group (Table 3).

Table 4 shows the prevalence of the ideal CVH metrics according to education and wealth index groups.

**Table 4:** Prevalence of ideal cardiovascular health metrics by education and wealth index groups, Serbia, 2013.

CVH metrics	Low education (n = 3080) % (95% CI)	Middle education (n = 6621) % (95% CI)	High education (n = 2045) % (95% CI)	P*	Poor wealth index group (n = 5043) % (95% CI)	Middle wealth index group (n = 2350) % (95% CI)	Rich wealth index group (n = 4353) % (95% CI)	P*
Ideal smoking	69.1 (67.4–70.8)	59.5 (58.3–60.6)	69.9 (67.8–72.0)	<0.001	64.0 (62.7–65.3)	63.7 (61.8–65.7)	63.6 (62.2–65.0)	0.928
Ideal healthy diet	0.7 (0.2–1.3)	2.2 (1.9–2.6)	5.4 (4.8–6.1)	<0.001	1.2 (0.8–1.6)	2.5 (1.9–3.1)	3.7 (3.3–4.2)	<0.001
Ideal physical activity	44.1 (42.3–45.8)	56.2 (55.0–57.4)	54.7 (52.5–56.8)	<0.001	52.0 (50.7–53.4)	52.1 (50.1–54.1)	53.9 (52.4–55.4)	0.151
Ideal BMI	32.6 (30.9–34.4)	42.1 (40.9–43.3)	46.5 (44.3–48.6)	<0.001	38.7 (37.4–40.1)	38.6 (36.6–40.5)	43.3 (41.8–44.8)	<0.001
Ideal blood pressure	9.7 (8.3–11.0)	19.4 (18.5–20.3)	23.5 (21.9–25.2)	<0.001	13.8 (12.8–14.8)	16.6 (15.0–18.1)	22.4 (21.3–23.5)	<0.001
Ideal cholesterol	82.4 (81.2–83.6)	88.1 (87.2–88.9)	86.7 (85.3–88.2)	<0.001	86.3 (85.4–87.3)	86.7 (85.3–88.1)	86.2 (85.2–87.2)	0.857
Ideal glucose	87.5 (86.5–88.4)	93.9 (93.3–94.6)	93.5 (92.4–94.7)	<0.001	90.9 (90.2–91.7)	92.0 (90.9–93.0)	93.7 (92.9–94.5)	<0.001

CVH, Cardiovascular health

BMI, Body mass index

*According to Chi-square test.

The ideal smoking status was more often present among respondents with high and low education than in those with middle one. An independent positive gradient was found between ideal healthy diet, ideal BMI, ideal blood pressure and education, i.e., more educated people had more often the above-mentioned ideal components. The ideal physical activity, ideal cholesterol, and ideal glucose were the most prevalent among respondents with middle educational attainment (Table 4).

The ideal healthy diet and socioeconomic status measured by the wealth index were in a positive, statistically significant relationship (Table 4). The smallest percentage of people who had ideal healthy diet belonged to poor wealth index group, while the largest percentage was from the rich one. The ideal BMI was the most prevalent among the rich wealth index group. The percentage of people with ideal blood pressure and ideal glucose increased with higher socioeconomic status. Concerning prevalence of other ideal CVH metrics (smoking, physical activity, and cholesterol), no statistically significant differences were found by wealth index groups (Table 4).

Table 5 shows the distribution of CVH metrics across sex and age groups. Only 0.1% of the Serbian population had all 7 CVH components at the ideal level, while the largest number had 4 and 3 CVH components. On average, women had more often 0, 1, 5, 6 and 7 ideal components of CVH than men (Table 5). Respondents from the youngest age group had more statistically significant 5, 6 and 7 CVH components at an ideal level in relation to those from the other two age groups (Table 5).

**Table 5:** Distribution of cardiovascular health metrics across sex and age groups, Serbia, 2013.

No. of ideal CVH metrics, % (95% CI)	Total sample (n = 11746)	Women (n = 6341)	Men (n = 5405)	P*	Age 20–39 (n = 3655)	Age 40–64 (n = 5535)	Age ≥65 (n = 2556)	P*
0	0.3 (0.2–0.4)	0.4 (0.3–0.5)	0.2 (0.1–0.4)	<0.001	0.0 (-0.2–0.2)	0.5 (0.4–0.7)	0.3 (0.1–0.5)	<0.001
1	3.0 (2.7–3.3)	3.2 (2.8–3.6)	2.7 (2.2–3.1)		0.5 (0.0–1.1)	3.7 (3.3–4. 1)	4.9 (4.2–5.5)	
2	13.6 (13.0–14. 2)	13.6 (12.7–14. 4)	13.6 (12.7–14. 5)		6.9 (5.8–8.1)	15.9 (15.0–16.8)	18.0 (16.7–19.3)	
3	31.4 (30.6–32.3)	29.2 (28.0–30.3)	34.1 (32.9–35.4)		23.4 (21.9–24.9)	34.2 (32.9–35.4)	37.1 (35.3–38.9)	
4	31.7 (30.9–32.6)	29.7 (28.6–30.9)	34.1 (32.9–35.4)		33.8 (32.3–35.3)	31.8 (30.6–33.0)	28.8 (27.0–30.6)	
5	15.8 (15.2–16.5)	17.8 (16.9–18.7)	13.6 (12.6–14.6)		26.4 (25.2–27.6)	11.6 (10.6–12.5)	10.0 (8.6–11.4)	
6	4.0 (3.7–4.4)	6.1 (5.6–6.5)	1.6 (1.1–2.1)		8.8 (8.2–9.4)	2.3 (1.7–2.8)	1.0 (0.2–1.7)	
7	0.1 (0.0–0.1)	0.1 (0.1–0.2)	0.0 (-0.1–0.1)		0.2 (0.1–0.3)	n.a.	n.a.	
6–7	4.1 (3.7–4.5)	6.2 (5.7–6.7)	1.6 (1.1–2.2)	<0.001	9.0 (8.3–9.6)	2.3 (1.8–2.8)	1.0 (0.2–1.7)	<0.001
2–5	92.6 (92.1–93.1)	90.2 (89.6–90.8)	95.4 (94.8–96.1)	<0.001	90.5 (89.7–91.4)	93.4 (92.8–94.1)	93.9 (92.8–94.9)	<0.001
0–1	3.3 (3.0–3.6)	3.6 (3.2–4.0)	2.9 (2.4–3.4)	0.036	0.5 (-0.1–1.1)	4.2 (3.8–4.7)	5.2 (4.5–5.9)	<0.001
No. of ideal health behaviors, % (95% CI)								
0	8.8 (8.2–9.3)	7.8 (7.1–8.5)	9.9 (9.2–10.7)	<0.001	8.0 (7.1–8.9)	10.8 (10.0–11.5)	5.5 (4.4–6.6)	<0.001
1	37.8 (37.0–38.7)	37.6 (36.4–38.8)	38.1 (36.9–39.4)		30.2 (28.6–31.7)	39.7 (38.4–41.0)	44.8 (42.9–46.7)	
2	39.1 (38.3–40.0)	39.0 (37.8–40.2)	39.3 (38.0–40.6)		40.9 (39.3–42.5)	38.8 (37.5–40.1)	37.3 (35.4–39.2)	
3	13.8 (13.2–14.5)	15.1 (14.3–16.0)	12.3 (11.4–13.3)		20.5 (19.4–21.7)	10.4 (9.5–11.3)	11.7 (10.3–13.0)	
4	0.4 (0.3–0.5)	0.5 (0.3–0.7)	0.3 (0.2–0.5)		0.4 (0.2–0.6)	0.3 (0.2–0.5)	0.7 (0.5–1.0)	
No. of ideal health factors, % (95% CI)								
0	0.7 (0.5–0.8)	0.8 (0.6–1.0)	0.6 (0.4–0.8)	<0.001	0.1 (-0.2–0.3)	1.1 (0.9–1.3)	0.7 (0.4–1.0)	<0.001
1	6.2 (5.8–6.6)	6.8 (6.2–7.4)	5. 5 (4.9–6.1)		1.2 (0.4–2.0)	8.4 (7.8–9.0)	8.5 (7.6–9.5)	
2	34.8 (33.9–35.6)	30.7 (29.5–31.8)	39.6 (38.3–40.8)		28.7 (27.2–30.3)	39.3 (38.1–40.6)	33.5 (31.7–35.3)	
3	49.3 (48.3–50.2)	48.7 (47.5–49.9)	49.9 (48.5–51.2)		51.2 (49.6–52.8)	45.5 (44.2–46.8)	54.6 (52.7–56.5)	
4	9.1 (8.6–9.6)	13.1 (12.4–13.8)	4.4 (3.7–5.2)		18.8 (17.9–19.7)	5.6 (4.9–6.4)	2.7 (1.6–3.7)	

CVH, Cardiovascular health

*According to Chi-square test.

The largest number of respondents had 2 ideal health behaviors, while all 4 behaviors had only 0.4% of the population, and this was significantly more common among women than men (Table 5). Regarding age groups, the prevalence of respondents with the simultaneous presence of all 4 ideal behaviors was the most frequent among the oldest, while 2 and 3 ideal behaviors were highest in the youngest respondents.

All 4 ideal health factors were present among 9.1% of the population, almost three times more likely in women than men and were highest in the youngest age group. Also, the prevalence of people with 3 ideal health factors was highest in the oldest age group (Table 5).

The results of the multivariable logistic regression analyses that assessed the association between demographic and socio-economic variables of the respondents and the number of ideal CVH metrics are presented in Table 6. The greater number of ideal CVH metrics in the population of Serbia was observed in women, younger people, people living without partner, more educated, as well as among the rich in relation to the poor (Table 6). The higher number of ideal health behaviors and ideal health factors was found in women, younger people, and more educated (Table 6).

**Table 6:** Association between cardiovascular health metrics and main demographic and socio-economic variables–multivariable logistic regression analyses, Serbia, 2013.

Variables	Ideal CVH metrics (6–7 vs. 0–5)		Ideal health behaviors (3–4 vs. 0–2)		Ideal health factors (4 vs. 0–3)	
	OR (95%CI)	*P*	OR (95%CI)	*P*	OR (95%CI)	*P*
Men	1		1		1	
Women	4.46 (3.52–5.65)	<0.001	1.35 (1.22–1.51)	<0.001	3.67 (3.15–4.27)	<0.001
Age 20–39	7.12 (4.64–10.93)	<0.001	1.49 (1.28–1.74)	<0.001	6.91 (5.29–9.03)	<0.001
Age 40–64	2.12 (1.36–3.30)	0.001	0.78 (0.67–0.91)	0.002	1.86 (1.41–2.45)	<0.001
Age ≥65	1		1		1	
Urban	1		1		1	
Rural	1.02 (0.80–1.29)	0.902	1.10 (0.97–1.26)	0.136	1.12 (0.95–1.32)	0.189
Married/living with partner	1		1		1	
Living without partner*	1.70 (1.40–2.06)	<0.001	1.36 (1.22–1.52)	<0.001	1.04 (0.90–1.20)	0.581
Low education	1		1		1	
Middle education	2.51 (1.72–3.67)	<0.001	1.68 (1.43–1.97)	<0.001	1.79 (1.44–2.23)	<0.001
High education	3.57 (2.36–5.40)	<0.001	2.46 (2.03–2.96)	<0.001	2.56 (1.99–3.30)	<0.001
Poor wealth index group	1		1		1	
Middle wealth index group	1.22 (0.91–1.63)	0.187	1.02 (0.88–1.19)	0.780	1.05 (0.86–1.28)	0.633
Rich wealth index group	1.43 (1.08–1.88)	0.012	1.12 (0.96–1.30)	0.141	1.25 (1.03–1.51)	0.021

CVH, Cardiovascular health; OR, Odds ratio; CI, Confidence interval

1, Reference value.

*Unmarried, divorced or widowed.

## Discussion

This is the first study to report information on the prevalence of ideal CVH from Serbia, and one of a few studies conducted in low- and middle-income countries. Our findings indicate that the prevalence of ideal CVH (ideal levels of all 7 CVH metrics) in the Serbian population is very low (0.1%). This prevalence estimate was the same or lower in comparison with several previous studies [19–26], but higher than in neighboring Republic of Srpska, Bosnia and Hercegovina (BH) [27] and in Shandon Province in China [28].

In recently performed comprehensive systematic review of 50 studies, ideal CVH (defining as achieving 6 or 7 ideal CVH metrics) ranged from as low as 0.3% in an Iranian study [29] to 15% in a large Chinese study [30], suggesting that the limited presence of ideal CVH is a global problem [7]. The prevalence of 6 to 7 ideal CVH metrics in our study was 4.1% like in studies from Spain [24] and Ecuador [25], but twice higher than in the study from BH [27].

The percentage of subjects who achieved the 4 ideal health behaviors was lower than the percentage of those with the 4 ideal health factors (0.4% vs.9.1%) that is in accordance with previous reports [21,24,27].

The prevalence of poor CVH (0–1 ideal CVH metrics) in our study was 3.3% that is higher in comparison with findings from the Chinese [31] and Korean [32] studies, but lower than in the most published studies on CVH [7].

Prevalence of ideal CVH metrics in the present study ranged from 2.4% for a healthy diet to 86.4%, and 92.2% for ideal levels of glucose and cholesterol, respectively. Despite different measurements, healthy diet was the poorest CVH metric in most studies included in the recently published systematic review on the prevalence of ideal CVH [7], except of two studies which used diet metrics not comparable with the AHA ideal metric for healthy diet [33,34]. Ideal blood pressure (17.5%) was the second poorest CVH metric, followed with ideal BMI (40.4%), and ideal physical activity (52.7%). Low frequencies of these ideal CVH metrics were observed across all the studies on CVH, but not always in the same order [7].

More than half of the Serbian population (63.8%) achieved ideal metric for smoking that is in accordance with nearly all studies included in the systematic review by Younus et al. [7] and could be mostly explained as the consequence of the worldwide tobacco use prevention efforts.

Our study revealed substantial disparities in CVH and CVH metrics by sex, age, and socio-economic factors.

Women had better ideal CVH than men (6.2% vs. 1.6%), and higher number of ideal CVH metrics. They achieved greater number of both, ideal health factors and ideal behaviors. These findings are consistent with a number of studies on CVH in general populations of high-income as well as in the middle- and low-income countries [21–24,26,27,30,35].

Like in previous studies [24,36] women were more likely to have ideal smoking, ideal healthy diet, ideal BMI and ideal blood pressure. Considering the epidemiological evidence that smoking is a stronger cardiovascular risk factor in women than in men [37] and increasing trend in female smoking, tobacco control policies in Serbia should particularly target women.

Better CVH (6–7 vs. 0–5 ideal CVH metrics) in the present study was observed in younger and middle-aged participants in comparison with the oldest age group. Those in the youngest age group had almost seven times a greater number of ideal health factors and a higher number of ideal behaviors than the oldest one. Our results are in accordance with findings from previous studies [21,24,27,28].

Ideal smoking and ideal healthy diet were the most prevalent ideal behaviors in the oldest age group, whereas ideal physical activity, ideal BMI, and cholesterol and glucose at ideal levels were the most prevalent in the youngest age group, which is similar to other studies [24,27].

In the available literature living alone has been inconsistently linked with CVH status. In the present study, living without a partner (unmarried, divorced or widowed) was associated with higher number of ideal CVH metrics that is in accordance with findings from neighboring Republic of Srpska, BH [27]. In contrary Del Brutto et al. [38] found a modest adverse effect of living alone on the CVH status and on some CVH metrics (physical activity, health diet, and glucose levels), as a consequence of living habits and lack of family support in those people. Recently Chang et al. [35] found that married/living with a partner had about two times better CVH than those living alone.

We found an independent positive gradient between education and CVH. Participants with high and middle educational levels had a significantly higher number of ideal CVH metrics, ideal health behaviors, and ideal health factors compared with those with low educational level. In several previous studies, participants with higher education had better CVH profile in comparison with low educated [22,24,35,39]. People with higher education were more likely to be employed and have more family income, which could enable them to improve living conditions and health behaviors. The prevalence of ideal healthy diet, ideal BMI and ideal blood pressure in the present study increased with higher education. In the Spanish study prevalence of ideal CVH metrics was higher in those with secondary or higher education except for smoking and healthy diet [24].

Higher numbers of ideal CVH metrics and ideal health factors were seen in the rich people compared to the poor ones. The prevalence of ideal healthy diet, and ideal blood pressure increased with higher socio-economic status measured by the wealth index. However, any significant association between the wealth index and CVH was not found in the neighboring Republic of Srpska, BH [39]. In the study conducted in Shandong Province in China, higher socio-economic status (higher education and income) was independently associated with an increased prevalence of meeting 5 or more CVH metrics in women but not in men [28].

When comparing the results of our study with other published studies on CVH, caution is recommended. Besides the differences in the age and sex composition of the populations and in the period over which data were collected, caution should also be taken on the different criteria for assessing ideal CVH (7 or 6–7 ideal CVH metrics) and poor CVH (0–1 or 0–2 ideal CVH metrics), and different data used for the assessment of CVH metrics. Whereas estimates of three health factors (blood pressure, cholesterol, and glucose) and calculation of BMI in studies from high-income countries were mainly based on direct laboratory, blood pressure and height and weight measurements [19,21], in the studies conducted in low- and middle-income countries, self-reported data were mainly used [26]. An exception is the study by Fang et al. that estimated status of CVH among the adult Americans in the 50 states and District of Columbia using self-reported data for all 7 AHA metrics from the Behavioral Risk Factors Surveillance System, the world's largest ongoing telephone health surveillance system [22]. In the present study, the combination of direct measurements and self-reported data was used. Data on hypertension and height and weight were obtained by direct measurements in the field, while self-reported data were used for the assessment of smoking, diet, physical activity, cholesterol, and diabetes.

The main strength of our study is the large representative sample of the Serbian adult population that allows assessment of CVH status to be generalized to the whole population. The most important limitation is that high cholesterol and diabetes that should be measured objectively [6] were instead determined by the participants' responses. As a consequence, the full range of the AHA categories of “ideal”, “intermediate” and “poor” CVH was not assessed. The presumed underestimates of high cholesterol and diabetes as the result of participants' lack of awareness, and smoking as undesirable behaviour, would most likely result in overestimates of ideal CVH in Serbian population. However, such underestimates are unavoidable [40] and were also seen in other studies that depend on self-reported data for CVH metrics [22,26].

Using the criteria recommended by AHA, we excluded those with self-reported coronary heart disease and stroke at the beginning of the study that would overestimate ideal CVH in the general Serbian population. In addition, residents in institutions and long-term facilities were excluded that also would overestimate ideal CVH. Besides, we were unable to study associations between CVH and CVD outcomes, since the cross-sectional study design prevents any conclusions regarding causality to be made.

In spite of all mentioned limitations the present study provides baseline assessment of ideal CVH and its predictors in the adult Serbian population. It is one of few studies conducted in developing countries, such as India [41], Iran [29], Republic of Srpska, BH [27], Ecuador [25], and Brazil [26], where the incidence of CVD is increasing.

Considering the strong association of CVH with CVD events [7,8,19], and the fact that CVH metrics are modifiable through lifestyle and treatment, a coordinated effort for improving CVH in Serbia at both individual and population levels should be a priority. Our results that only a minority of Serbian population meets criteria for ideal CVH, especially for health behaviors, provide enormous room for improvement. Successful prevention efforts that improve healthy diet and physical activity should result in an improvement in BMI, blood pressure, and glucose level, and even modest changes will result in substantial cardiovascular benefits.

Our findings of existing age-specific, sex and socio-economic differences in the prevalence and number of ideal CVH metrics should serve for the development of appropriate CVD prevention policies tailored to fit specific needs of both sexes, all age groups and people with different socioeconomic status.
